# Unconscious overtone manipulation and transmission in flute performance: insights into musical expression and perception

**DOI:** 10.3389/fpsyg.2025.1393689

**Published:** 2025-02-19

**Authors:** Kai Hiraiwa, Masanobu Miura

**Affiliations:** ^1^Graduate School of Music, Kunitachi College of Music, Tokyo, Japan; ^2^Faculty of Music, Kunitachi College of Music, Tokyo, Japan

**Keywords:** flute timbre, overtone structure, principal component analysis (PCA), listening experiments, musical expression, auditory perception

## Abstract

This study aims to elucidate the overtone structure of the flute, examine the impact of acoustic parameter alterations on timbre perception, and foster a shared vocabulary among players and listeners. Employing principal component analysis (PCA) and listening experiments, the investigation delves into the ways in which players subconsciously adjust timbre and the manner in which these adjustments are perceived by listeners. The analysis concentrated on overtone components up to the fifth overtone (5*f*_0_) utilizing flute long-tones. PCA revealed that the first principal component (PC1) predominantly captured variations in the overall strength of overtones, whereas PC2 and PC3 were indicative of a balance around specific overtones. Furthermore, the coordinates of PC1, PC2 and PC3 for sounds deliberately produced with varying timbres were found to diverge. This finding indicates that the flute's timbre is influenced by both the overall loudness and the balance of overtones, and that players modify the overtone structure for expressive purposes. A listening experiment involving 28 participants ascertained that listeners were capable of distinguishing between different timbres, revealing significant differences in the percentage of coincident judgment based on the player and the musical register. Notably, professional players were more adept at conveying the intended timbre to the listener, and the middle register was identified as having the greatest potential for expressive variation.

## 1 Introduction

Music encompasses a myriad of values predominantly resting on subjective evaluations and verbal expressions rooted in impressions. The complexity of impressions received from musical performances intertwines with multifarious factors, rendering it challenging for listeners to precisely articulate why they perceive a performance as “good” or otherwise.

In classical music, a field in which the first author specializes, a unique structure exists where various players perform the same musical piece repeatedly, eliciting preferences from the audience based on slight differences attributable to the performers' individuality from the same score. Such distinctions enable the audience to categorize performances according to preference or the lack thereof. Moreover, variations in the performances of the same musician across different pieces or even the same piece at different ages are recognized as distinct, reflecting the nuanced nature of musical performance.

Nevertheless, the specific differences between these performances often remain elusive, with assessments of sound or performance improvement largely subjective to the listener. Neither the listener nor the performer can definitively identify which changes in acoustic parameters contribute to these perceived differences.

Recent efforts have been made to estimate proficiency in instrumental performance (Nonogaki et al., 2011; Okemoto and Miura, 2020), suggesting that performances of the same musical piece by players of similar proficiency can still be distinct in content and evaluation. Prior studies have modeled the flute's instrument structure (Coltman, 1968; Ando, 1969; Terrien et al., 2013; Fletcher and and Rossing, 2012), including measurements of lip shape and the distance from the lips to the apex (de la Cuadra et al., 2008), and explored the relationship between exhalation and sound production (Lefebvre and Scavone, 2012). Controlling multiple performance parameters facilitates the transmission of emotion through music, allowing the performer to deviate from the composer's indications by employing elements not specified in the score, such as timbre (Juslin, 2000). Contrastingly, for instruments such as the clarinet and oboe, experiments have been conducted to discern and distinguish timbres (Saldanha and Corso, 1964).

In a previous study by the authors, a distinction was made between the long-tones practiced by wind instrument players as a fundamental exercise and the notes employed in actual musical performances. This work emphasized differences in overtone structure through the Mahalanobis distance (Hiraiwa and Miura, 2023), focusing on musical phrases requiring minimal dramatic changes and similar nuances. Five partials were identified from the first overtone (*f*_0_) to the fifth (5*f*_0_), with quantification on the compositional balance of these overtones. Utilizing a Vector Autoregression Model, it was demonstrated that players unconsciously adjust the balance of overtones to maintain consistent musical expression by referencing their previous performances of a piece (Hiraiwa and Miura, 2023). This unconscious control over the transition of overtone balance is hypothesized to ensure smooth and convincing musical performances.

Vocalists and wind instrumentalists, unable to hear their sound from the listener's perspective throughout their careers, continuously compare their sound with third-party impressions to enhance their technique. Through intersubjective matching, they can only conjecture how their sound might be perceived in an auditorium.

Players have individual methods for improving their performance; nonetheless, such enhancements are subjectively aligned with their ideals. For instance, a player seeking a warmer sound might experiment with various techniques to achieve this effect, relying on empirical understanding rather than direct manipulation of specific overtones.

This research aims to visualize the sound structure of the flute and the transitions of acoustic parameters during performance, ultimately developing parameters that reflect musical quality and establish a common language for listeners and players. Specifically, it will examine changes in the flute's timbre made unconsciously by the player as part of their musical expression and how these changes are perceived by listeners through listening experiments. This study is not about the perception of timbre and its linguistic transmission, rather the generation of common understanding in the perception of timbre. While certain studies have investigated sounds deemed “good” by skilled flutists and the impact of listener proficiency (Kasahara et al., 2018; Yorita and Clements, 2015), judgments of relative sound quality among players with considerable experience often remain subjective. In this study, timbre, which is essential in musical expression, was analyzed in terms of the type of changes in the balance of overtones responsible for the tonal differences that players currently rely on in their subjective expressions (von Bismarck, 1974a,b; Saitis and Weinzierl, 2019). Furthermore, it investigates whether listeners can discern the changes in timbre as the player intends them to be, in response to a performance in which the player has changed the timbre.

The potential for players to recognize their performance improvements through visual information and numerical values, transcending the limitations of verbal expressions such as “better” or “improved”, presents a significant advancement. It enables a tangible representation of performance differences accessible to the performers themselves.

## 2 Materials and methods

The study is structured into two main sections:

1. The first section employs principal component analysis (PCA) (Hotelling, 1933; Hastie et al., 2001) to dissect the timbre changes in flute music, illustrating that these changes are primarily due to variations in the balance of overtones. This analysis aims to ascertain which elements of the overtone series are predominantly affected by shifts in their balance.
2. The second section delves into understanding how listeners perceive the timbre changes identified in the first section through listening experiments. It examines the impact of the player's musical expression on timbre and which overtone elements are significantly influenced by changes in the balance of overtones. Furthermore, it explores whether the timbre changes controlled unconsciously by the player are perceived by listeners as the player intended, based on the results of the PCA analysis.

### 2.1 Sound source

In this study, we analyzed the flute's tonal characteristics by recording long-tones and tones at the same pitch but with varied timbres within the instrument's playable range. We focused on the difference in the number of components for each harmonic, ranging from the fundamental frequency (*f*_0_) to five times the fundamental frequency (5*f*_0_). This range was chosen as the parameter group for conducting PCA (Pearson, 1901). The methodology for calculating specific parameter values is detailed below.

All sound recordings were captured at a sampling frequency of 48,000 Hz with a 16-bit depth. From these recordings, 16,384 points were extracted for analysis. A fast Fourier transform (FFT) was applied using a Hanning window, and the power spectrum was calculated, setting the energy of *f*_0_ at 0 dB.

The decision to limit our focus to the 5*f*_0_ overtone is based on the flute's extensive playable sound range, which allows for stable and reliable partial observations up to 5*f*_0_. This approach is consistent with similar studies on instruments with comparable structures to the flute, such as recorders, where observations up to the 5*f*_0_ have been employed to investigate changes in timbre (Haverkamp, 2023).

For the purpose of this analysis, flute performances were recorded by three flutists, including the first author. Table 1 provides details regarding each player and the instruments utilized in the study.

**Table 1:** Details of each flutist and the instrument.

**Player**	**Details of instruments**	**Flute experiences**	**Details of player**
X	Altus 1807AL REH	33 years	Associate professor at a music college and former professional orchestra player
Y	Altus A14K(S)II REH	19 years	Student of music college in Doctor course
Z	Altus 1807AL REH	13 years	Master's degree at a music college

A total of 37 notes, spanning the flute's playable range from C4 to C7, were analyzed. This range encompasses three registers: the low register from C4 to B4, the middle register from C5 to B5, and the high register from C6 to C7. The recording of all tones was conducted in stereo using two microphones: an ambient microphone (RODE NT2-A), positioned 238 cm from the flute, and a proximity microphone. These recordings were subsequently monoised at a ratio of 1:1 for the purpose of analysis. The observed difference in loudness between the proximity and ambient recordings is approximately 8.14 dB. The recording was conducted in a lesson room at the Kunitachi College of Music, with an RT60 of 0.0273 s.

### 2.2 Respondents

In total, 28 respondents (22 women and 6 men) completed the survey. The participants primarily comprised university and postgraduate students who speak Japanese, with ages ranging from 18 to 48 years and an average age of 25.75 years. The survey aimed to collect a sample from individuals who listen to music daily, thereby being regularly exposed to and contemplative of the differences in instrumental timbre. Responses were primarily solicited from our universities and their affiliates. Of these respondents, 26 are either currently enrolled in or have graduated from music colleges, with 14 of them specializing in flute.

### 2.3 Stimuli and procedure

#### 2.3.1 Long-tone

To elucidate the fundamental overtone structure of the flute's performance sound and compare it with sounds where the timbre has been altered, with the objective of enabling players to sustain a “good sound” to the greatest extent possible during long-tone playing, recordings were made of long-tones played by three individuals. Three beats at a tempo of 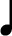 = 60 were recorded for each note from C4 to C7.

Each peak power spectral was averaged separately to analyse the harmonic structure of the long tones. Specifically, the average values of *f*_0_ to 5*f*_0_ were used in the analysis. To explore variations in timbre, five patterns of long-tones, lasting 1 s for each note, were generated by altering the starting second of the excerpt in five predetermined locations within the specified range. To remove the effects of tonguing, five sample patterns were obtained by using tones within 0.5–2.5 s of the three seconds, which involved shifting the starting time by 0.25 s and cutting out 1 s samples.

#### 2.3.2 Performance sound with different timbre

While long-tones are foundational to instrumental performance, musicians often employ tones diverging from the “good tones” associated with long-tones to enhance musical expression. Given the infinite variety of tonal patterns that can emerge from the piece's content and the performer's interpretation, this study introduced sound nuances encapsulated by two sets of opposing tones: “quiet (p)/intense (f)” and “cold/warm”. The objective was to execute the combination patterns of sounds detailed in Table 2. Participants X, Y and Z attempted to embody the timbre characteristics to the extent feasible according to their personal interpretation. They permitted themselves unlimited re-recordings until achieving satisfaction with the sound quality of their performances, focusing on the notes A4, A5, and A6.

**Table 2:** Patterns of timbre changing.

	**Cold**	**Warm**
Quiet (p)	(i)	(ii)
Intense (f)	(iii)	(iv)

#### 2.3.3 Listening experiments with tones of changing timbre

As previously mentioned in Section 2.3.2, two recordings of each of the four timbre patterns were made. However, listening experiments for quiet and intense timbres were conducted separately due to the significant difference in sound pressure levels between the two. In the listening experiment focusing on the differentiation between (cold, p) and (warm, p) in quiet tones, two recordings of (cold, p) and two of (warm, p) at the same pitch and performed by the same player were randomly rearranged. Listeners were subsequently asked to make a choice in a two-alternative forced choice format (Bogacz et al., 2006), deciding whether the four sounds were relatively “(more or less) cold” or “(more or less) warm”. In this case, specifying two sounds as “cold” and two as “warm” was unnecessary; participants instead assessed all sounds, aiming to classify them as “warm” to the greatest extent possible.

**Figure 1:**
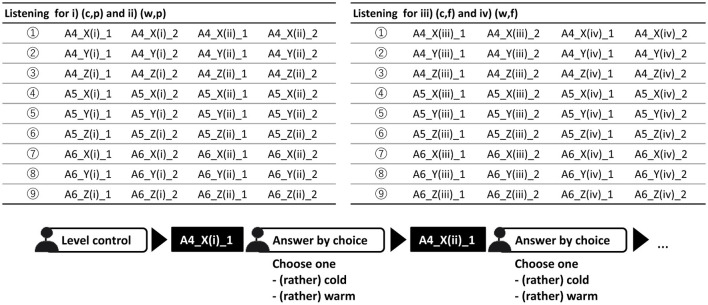
Flowchart of the experiment.

This procedure was replicated for all players and pitches. Figure 1 presents the labels of all sounds used in the listening experiment and provides an example of the experimental flow. Each listener was tasked with evaluating 72 sounds.

Notational example: Two cuts of A4 (cold, p) tone played by X is A4_X(i)_1, A4_X(i)_2, A4 (warm, p) tone played by X is A4_X(ii)_1, A4_X(ii)_2,… etc.

### 2.4 Ethical note

This study received approval from the Ethical Committee of Kunitachi College of Music, Faculty of Music Research, under approval number 2339 on 13 December 2023. Conducted in alignment with the Declaration of Helsinki, all participants provided informed consent for their participation in the study and for the processing of their personal data.

### 2.5 Statistical analysis

PCA was conducted on the overtone components ranging from 2*f*_0_ to 5*f*_0_, with the exclusion of the fundamental frequency (*f*_0_) and the parameters used to calculate the difference in the number of components across the overtones. The 10 parameters employed in this analysis are detailed in Table 3.

**Table 3:** Parameters of PCA.

**Name**	**Details**
D_1 − 2_	Difference between F0 and 2F0 (F0–2F0)
D_1 − 3_	F0–3F0
D_1 − 4_	F0–4F0
D_1 − 5_	F0–5F0
D_2 − 3_	2F0–3F0
D_2 − 4_	2F0–4F0
D_2 − 5_	2F0–5F0
D_3 − 4_	3F0–4F0
D_3 − 5_	3F0–5F0
D_4 − 5_	4F0–5F0

Binomial and chi-square tests are used to analyse the results of the listening experiment.

## 3 Results

### 3.1 Long-tone and performance sounds with different timbres

**Figure 2:**
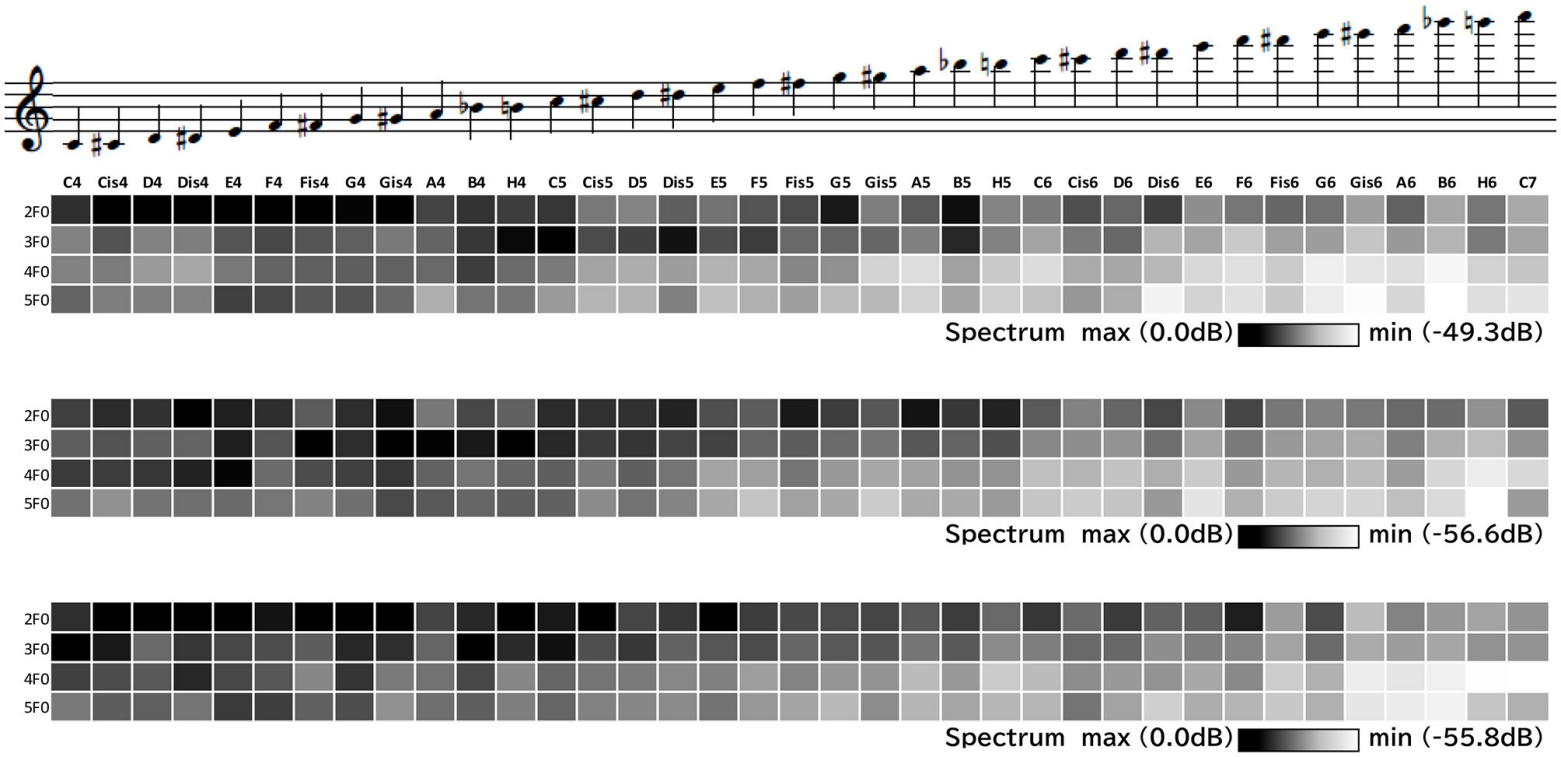
2*f*_0_-5*f*_0_ component amount in long-tones (X, Y, Z from the top).

The distribution of the harmonic components 2*f*_0_, 3*f*_0_, 4*f*_0_, and 5*f*_0_ for the five long-tones is presented in Figure 2.

**Figure 3:**
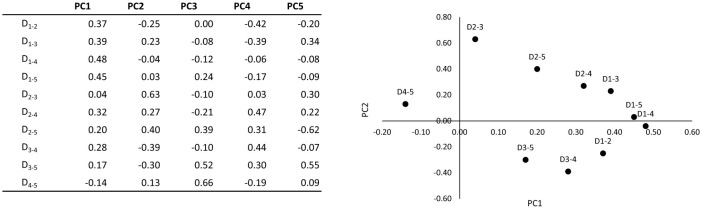
Loading plot of PC1 vs. PC2.

**Figure 4:**
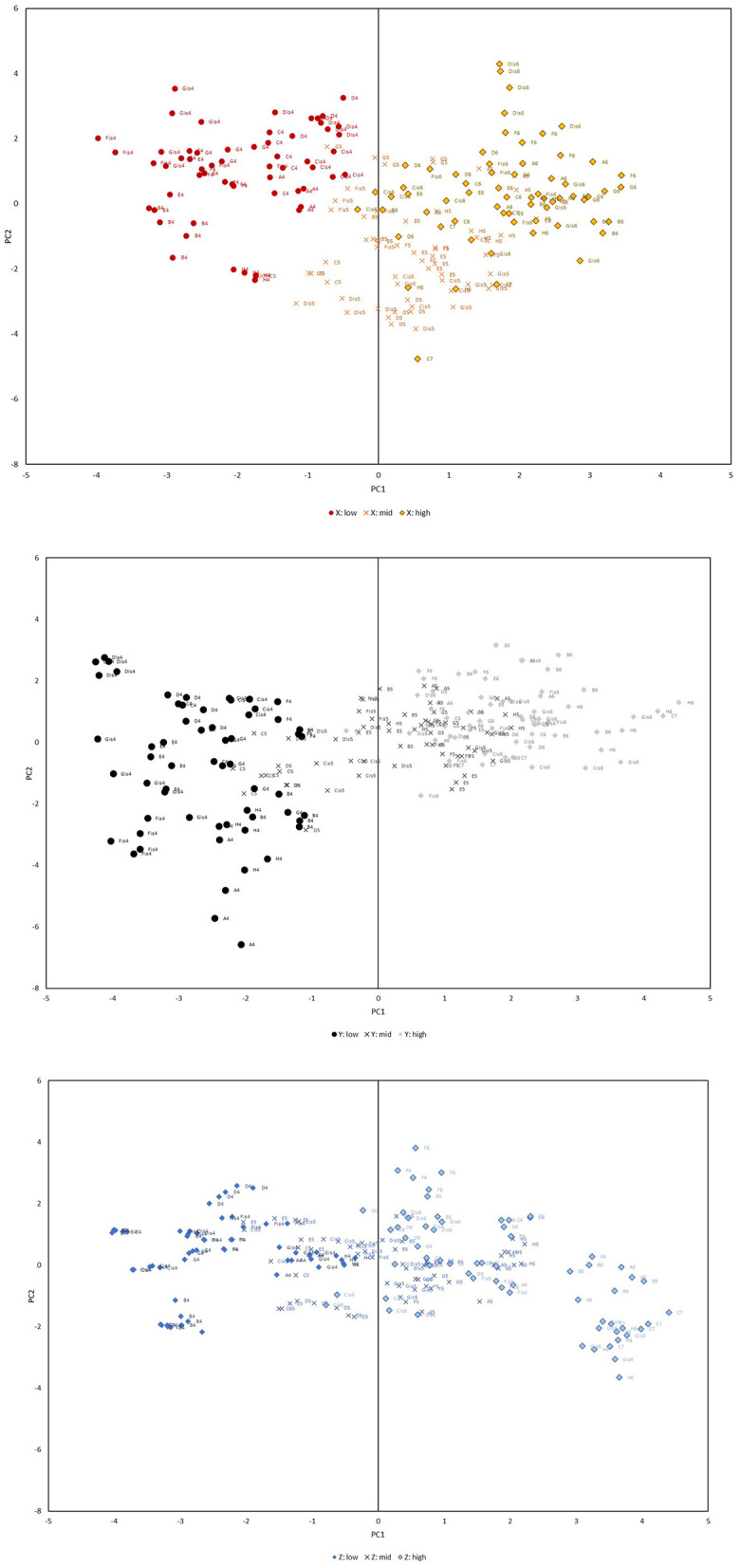
Long-tone distribution of the three players.

The results of PCA performed on five patterns of long tone data for each of the three players, utilizing the parameters specified in Table 3, are presented in Table 4. This table shows all Standard Deviations (equal to the square root of Eigenvalues) up to the fifth principal component, contribution rates, and eigenvectors for each variable. Figure 3 depicts a loading plot with the first principal component (PC1) and the second principal component (PC2). In addition, a plot of PC1 and PC2 for each long tone, separately for players X, Y and Z, is shown in Figure 4. Although there was variation according to pitch, a tendency for the range to increase as PC1 progressed in the positive direction was observed for all players. Furthermore, as indicated in Table 4, the cumulative contribution rate of the third principal component (PC3) reached 0.85.

**Table 4:** Summary of each principal component and principal component loadings.

	**PC1**	**PC2**	**PC3**	**PC4**	**PC5**
Standard deviation	2.03	1.58	1.38	1.21	0.00
Proportion of variance	0.41	0.25	0.19	0.14	0.00
Cumulative proportion	0.41	0.66	0.85	1.00	1.00
D_1 − 2_	0.37	−0.25	0.00	−0.42	−0.20
D_1 − 3_	0.39	0.23	−0.08	−0.39	0.34
D_1 − 4_	0.48	−0.04	−0.12	−0.06	−0.08
D_1 − 5_	0.45	0.03	0.24	−0.17	−0.09
D_2 − 3_	0.04	0.63	−0.10	0.03	0.30
D_2 − 4_	0.32	0.27	−0.21	0.47	0.22
D_2 − 5_	0.20	0.40	0.39	0.31	−0.62
D_3 − 4_	0.28	−0.39	−0.10	0.44	−0.07
D_3 − 5_	0.17	−0.30	0.52	0.30	0.55
D_4 − 5_	−0.14	0.13	0.66	−0.19	0.09

From Table 4, Figure 4, the first principal component (PC1) primarily reflects the magnitude of each harmonic's distance from the fundamental frequency (*f*_0_), specifically from 2*f*_0_ to 5*f*_0_. This suggests that PC1 correlates significantly with the strength of 2*f*_0_, 3*f*_0_, 4*f*_0_ and 5*f*_0_, with an increase in PC1 associated with a rise in the fundamental frequency across lower to higher frequencies. Consequently, all overtone components exhibit augmentation as the fundamental frequency escalates from lower to higher registers.

Conversely, the second principal component (PC2) emerges as crucial, with positive loadings on 2-3, 2-4 and 2-5, and negative loadings on 3-4 and 3-5. This indicates that if the balance among 2-3-4, 2-3-5, 2-4 and 2-5 is deemed essential, PC2 may play a role in the amalgamation of overtones cantered around 2*f*_0_. Alternatively, by examining the positive and negative loadings, the reversed signs for 2-3 vs. 3-4 and 2-3 vs. 3-5 suggest a reliance on the strength of 3*f*_0_, aligning with the perfect fifth relative to *f*_0_. Thus, PC2 is implicated in modulating the overtone balance, contrasting with PC1′s influence on the overall overtone intensity.

Moreover, the third principal component (PC3) features significant 3-5 and 4-5 elements, indicating a focus on the balance of 5*f*_0_, which aligns with the major third relative to *f*_0_.

**Figure 5:**
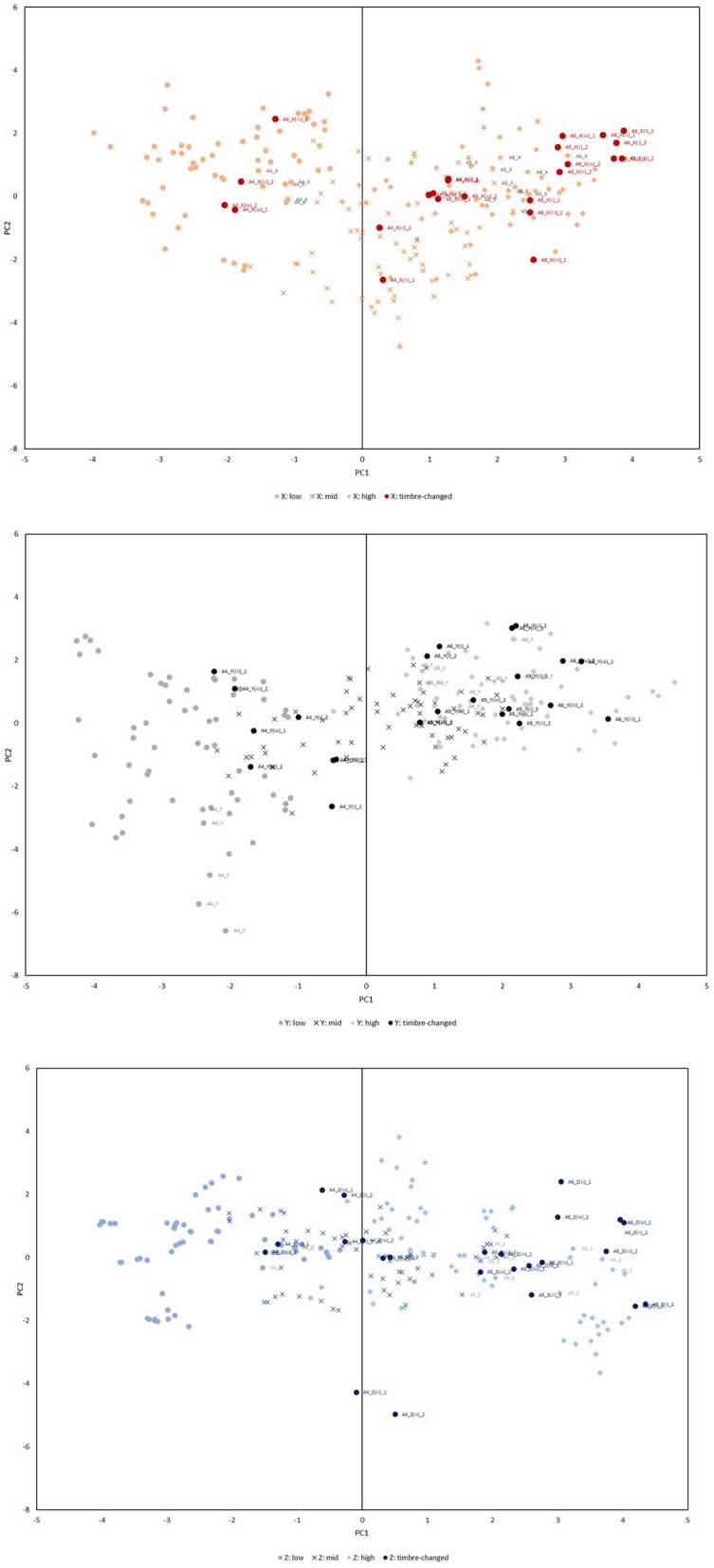
Mapping of the distribution of timbre-changed A4, A5, and A6.

For the analysis, two segments (3s each) matching the successfully performed long-tones were prepared for each pattern, and the overtone structure was analyzed similarly to ascertain PC1, PC2 and PC3, which were subsequently juxtaposed with the long-tones of identical pitch. Figure 5 supplements this with an additional plot of the four timbre patterns, distinguishing long-tone plots in a subdued shade as shown in Figure 4.

Alterations in timbre resulted in scattered instances where the sound substantially diverged from the PCA coordinates of a long-tone of the same pitch. This deviation among the PCA coordinates across the four patterns highlights the performer's subconscious modulation of the overtone balance to facilitate a wider expressive range. This phenomenon was similarly observed across the PC1-PC3 and PC2-PC3 axes.

In this study, each player recorded four patterns, with two takes (cuts) for each pattern, across the notes A4 to A6. To assess the similarity and differences in timbre among these recordings, three specific comparisons were made for each cut:

1. Comparison with the other cut recorded as the same tone (to assess consistency within the same timbre).
2. Comparison with a cut that shares the same “quiet/intense” attribute but differs in “cold/warm” attribute (to examine the impact of changing one aspect of timbre while keeping the other constant).
3. Comparison with another cut recorded with the same timbre as in point 2 (to validate consistency within the changed timbre).

Notably, the exact differences in how the listener perceives the change in timbre include those attributable to instrumental differences.

The study utilized the Euclidean distance in the 3D space defined by the first three principal components (PC1, PC2 and PC3) to quantify the differences between these cuts. This approach led to the observation of a significant difference in the average distances between sounds of the same timbre compared with those of different timbres, with a statistical significance (*p* < 0.01).

Table 5 in the study likely presented the detailed correspondence between each tone and the mean distances for each category, although this table was not displayed here. The results of the Friedman test, which compared the mean distances across the three groups mentioned above, confirmed the significance of the differences between these means. The findings indicate that the distance between cuts of the same tone was markedly shorter than between cuts of different tones, suggesting that both the overall magnitude of the overtones and their balance play a crucial role in timbre perception.

**Table 5:** Correspondence between each tone and the mean distances in each category.

				**Distance from base tone**		
**Base tone**	**(1)**	**(2)**	**(3)**	**(1)**	**(2)**	**(3)**
A4_X(i)_1	A4_X(i)_2	A4_X(ii)_1	A4_X(ii)_2	0.064	0.781	3.221
A4_X(i)_2	A4_X(i)_1	A4_X(ii)_1	A4_X(ii)_2	0.064	0.718	3.153
A4_X(ii)_1	A4_X(ii)_2	A4_X(i)_1	A4_X(i)_2	1.659	3.002	4.411
A4_X(ii)_2	A4_X(ii)_1	A4_X(i)_1	A4_X(i)_2	1.659	3.725	3.033
A4_Y(i)_1	A4_Y(i)_2	A4_Y(ii)_1	A4_Y(ii)_2	3.975	5.784	4.427
A4_Y(i)_2	A4_Y(i)_1	A4_Y(ii)_1	A4_Y(ii)_2	3.975	3.868	4.449
⋮	⋮	⋮	⋮	⋮	⋮	⋮
A6_Z(iv)_2	A6_Z(iv)_1	A6_Z(iii)_1	A6_Z(iii)_2	0.966	2.687	2.844
Average				1.024	3.064	3.41

This significant finding highlights that players are capable of altering timbre by manipulating the overtone structure in response to the specific imagery or descriptive language used (e.g., “quiet/intense” and “cold/warm”). It reflects the nuanced control musicians have over their instrument's sound production, allowing them to adjust their playing technique to convey different emotional and sonic characteristics intentionally.

### 3.2 Results of listening experiments

**Figure 6:**
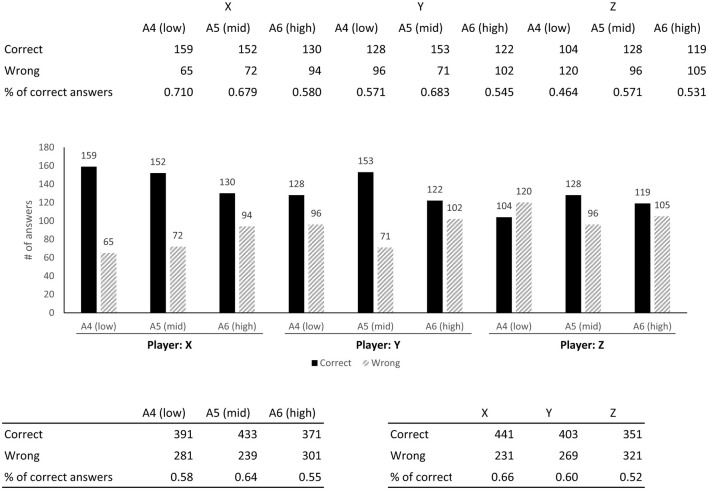
Results of responses to the listening experiment.

The aggregated outcomes for all 28 responses are shown in Figure 6. A response is deemed correct if the listener identifies a cold tone played by the player as “(rather) cold”. This criterion equally applies to warm tones. The results of the responses facilitate the rejection of the null hypothesis via a binomial test (*p* < 0.01).

Furthermore, a chi-square test was conducted to examine the number of correct and incorrect responses. The analysis revealed a significant difference in the percentage of correct responses between the player and register (*p* < 0.01). Conversely, the relevance of the respondent's specialization in flute to the accuracy of responses was found to be statistically insignificant (*p* = 0.821).

## 4 Discussion

### 4.1 Principal component analysis

The observation that the first principal component (PC1) encapsulates the overall increase or decrease in the overtone components and demonstrates a level of cohesion by the register in which the flute can be played aligns logically with the structural design of the flute. Specifically, it appears plausible that the overtone structure is categorized by register to a certain extent, given that the flute's playable registers are organized such that the fingering patterns loop with each octave increase.

Despite the evident indication that PC2 and PC3 are associated with the balance around 2*f*_0_, 3*f*_0_ and 5*f*_0_, the flute, being an instrument capable of producing very high notes, presents challenges in discerning overtones by ear during performance. Unlike the piano, where resonant strings are visible, it becomes complex for a player to intentionally emphasize the 3*f*_0_ overtone, which aligns with the perfect fifth degree, with the objective of enhancing its volume. Nevertheless, in practice, players do manipulate these overtones to convey musical expressions. The PCA illustrates that the coordinates not only diverge significantly between sounds of different timbres but also between long tones of the identical pitch.

### 4.2 Listening experiments

The percentage of correct answers per player was the highest for Player X, who was distinguished as the sole professional among the three participants in this session. The variance in instrumental proficiency reveals a key insight: higher skill levels correlate with an enhanced capacity to modulate the instrument. To enable listeners to accurately perceive changes in timbre, not only should the instrument produce a quality sound but also demonstrate a broad spectrum of tonal variation. Hence, it logically follows that more adept players are better equipped to convey a timbre that aligns more closely with their intentions to the listener.

Regarding the percentage of correct responses per register, the highest accuracy was observed in the middle A (A5), which insightfully mirrors the structural characteristics of the flute. Unlike other woodwind instruments, the flute lacks an octave key, relying instead on the manipulation of breath speed and pressure to control its tonal range. Typically, lower notes require a slower breath, whereas higher notes demand a swifter breath. Consequently, an excessive increase in breath speed when playing lower notes inadvertently shifts the note into the middle register, similar to how an undue decrease in breath speed when playing higher notes causes the note to drop into the middle register.

Thus, the flute most readily accommodates a diverse range of performance modifications within the middle register, achieving optimal sound quality. This finding concurs with the results of this experiment. It should be noted that it is premature to conclude that more skilled players are better at blowing tones. The sample of performers needs to be further increased. In addition, it cannot necessarily be asserted that the percentage of correct responses increases with increasing proficiency, as the analysis did not include the sounds played by novice players. Alternatively, more extreme expressions are possible for beginners. However, it is also true that these are the types of tones that are rarely used when actually performing in public.

## 5 Conclusion

In this study, PCA was conducted on long-tones and altered performance sounds, focusing on overtones and the variation in component numbers among these overtones as parameters. The overtone structure of the flute is partially dependent on pitch; however, by modifying the overtone structure at a consistent pitch, a variety of expressions can be imparted to the flute. The PCA utilized PC1, PC2 and PC3 to calculate the distances between performance sounds of identical timbres and those of differing timbres, revealing that the distances between sounds of the same timbre are notably shorter.

The outcomes of the listening experiment indicated that listeners could effectively distinguish between “cold” and “warm” tones. It was also observed that the rate of correct responses varied significantly based on the player and the tonal range. Professional players demonstrated a higher proficiency in communicating the intended tone to the listeners. Additionally, listeners most accurately discerned differences in expression within the middle register, which is where flute players typically find it most comfortable to play.

These findings imply that the timbre of the flute is shaped by the overall loudness and the balance of overtones, with players adjusting the overtone structure for expressive intentions. Such manipulations of overtones are conducted unconsciously by the player, who alters the timbre based on auditory judgment. When changing timbre, players do not consciously aim to produce a different overtone structure; rather, they inadvertently select sounds with a distinct overtone structure. The deviation from the overtone structure of long tones also indicates that the absence of a singularly “correct” or “ideal” overtone structure, suggesting that diverse overtone structures facilitates a spectrum of musical expressions.

Accurately conveying the intended timbre to the listener requires a high level of performance skill in reproducing the suitable overtone structure, influenced, to a certain degree, by the instrument's characteristics.

Future research should address three primary concerns:

1. To ascertain how proficiency with the instrument affects the production of different tones, an analysis involving the recorded performance sounds of both professional players and novices who have recently begun playing the instrument is necessary.
2. Additional factors indirectly related to timbre but potentially influencing the listener's perception, such as breath noise inherent to the flute's structure and vibrato, must be considered.
3. Enabling players to verify the overtone structure of the concerned tone upon alteration could facilitate an objective assessment and enhance control over musical expression. Implementing a real-time sound measurement system represents a significant challenge in achieving this goal.

## Data Availability

The original contributions presented in the study are included in the article/supplementary material, further inquiries can be directed to the corresponding author.
